# Thermophilic Fungi as the Microbial Agents of Choice for the Industrial Co-Fermentation of Wood Wastes and Nitrogen-Rich Organic Wastes to Bio-Methane

**DOI:** 10.3390/microorganisms11102600

**Published:** 2023-10-21

**Authors:** John G. Ingersoll

**Affiliations:** ECOCORP INC., 1211 South Eads Street, Suite 803, Arlington, VA 22202, USA; jgingersoll@ecocorp.com; Tel.: +1-202-999-0940

**Keywords:** fungus, hydrolysis, fermentation, thermophilic, wood, bio-methane, microbiome

## Abstract

The novel industrial approach of co-fermenting wood wastes with agricultural wastes that are rich in nitrogen such as animal manures to produce bio-methane (renewable natural gas) fuel via thermophilic anaerobic digestion mimics an analogous process occurring in lower termites, but it relies instead on thermophilic fungi along with other thermophilic microorganisms comprising suitable bacteria and archaea. Wood microbial hydrolysis under thermophilic temperatures (range of 55 °C to 70 °C) and aerobic or micro-aerobic conditions constitutes the first step of the two-step (hydrolysis and fermentation) dry thermophilic anaerobic digestion industrial process, designated as “W2M3+2”, that relies on thermophilic fungi species, most of which grow naturally in wood piles. Eleven thermophilic fungi have been identified as likely agents of the industrial process, and their known growth habitats and conditions have been reviewed. Future research is proposed such that the optimal growth temperature of these thermophilic fungi could be increased to the higher thermophilic range approaching 70 °C, and a tolerance to partial anaerobic conditions can be obtained by modifying the fungal microbiome via a symbiotic existence with bacteria and/or viruses.

## 1. Introduction

Woody biomass represents an abundant renewable resource that is carbon-neutral by design, and consequently, it has been a topic of intense interest as a source of renewable energy. However, the traditional approach of the combustion of wood to extract energy in a large scale is no longer considered to be an environmentally viable choice [1]. Thus, other pathways for the utilization of woody biomass must be developed. One such pathway is based on the industrial replication of naturally occurring processes observed in lower termites [2]. This pathway has been converted into an industrial process designated as “Wood to Methane 3+2” and abbreviated as “W2M3+2” [3,4]. This process consists of the microbial fermentation or anaerobic digestion of wood wastes into bio-methane as an advanced transportation fuel with a carbon-negative footprint, along with the generation of other co-products such as commercial grade phosphate, nitrogen and potassium bio-fertilizers, and green carbon dioxide to be used as either an industrial gas or a feedstock for other chemicals and fuels including additional or secondary bio-methane production with the aid of hydrogen generated via water electrolysis using wind and solar power. A major departure of the industrial conversion process from the natural one occurring in lower termites is that the former is designed to operate in the thermophilic temperature range of 55 °C to 70 °C, unlike the latter, which is of a mesophilic nature with optimal operating temperatures below 40 °C. Thus, a different set of microbes, namely, thermophilic fungi, is employed to promote the fermentation of wood. Otherwise, the industrial process is quite analogous to the natural one that is observed in lower termites. In the next section, brief descriptions of the natural degradation of wood to bio-methane using lower termites is given along with the industrially developed biological process used to convert wood to bio-methane. In the subsequent sections, the various thermophilic fungi that are potentially associated with the conversion process of industrial wood to bio-methane are considered in some detail, and lastly, research recommendations are made to enhance the conversion efficacy of these fungi in the future to further optimize the aforementioned industrial conversion process.

## 2. Naturally Occurring and Industrially Based Wood Co-Digestion to Bio-Methane

As is well known, wood is highly resistive to microbial attack such that its natural decomposition process can be accomplished effectively via a complex symbiotic natural process comprising several organisms, some macro- and mostly microorganisms, and is observed in the exclusively wood-feeding lower termites [2]. The natural process consists of three major steps that can be simplified as follows: (a) a mechanical size reduction in the wood into millimeter-sized particles by the insect to increase its surface area such that the other symbionts living in the termite have an increased access to the cellulose and hemicellulose components, with the insect thereby serving as the natural bioreactor vessel; (b) the assimilation of the small wood particles via eukaryotic single-cell organisms comprising flagellate protozoa (*Parabasalia* and *Preaxostyla* phyla) to hydrolyze cellulose and hemicellulose sugars into short-chain fatty acids and acetate; and (c) the fermentation of fatty acids and acetate into methane as well as the mineralization of nitrogen from the environment into ammonia via bacteria (mostly *spirochaetes* but also *bacteroides*, *firmicutes*, *proteobacteria*, and others) and archaea (various types of *methanogens*), with the ammonia being recycled back into the insect and the protists to supplement their nitrogen diets. The insect bioreactor releases methane and carbon dioxide along with lignin-rich feces. The termite bioreactor is very efficient as it removes between 74% and 99% of cellulose and 65 to 87% of hemicellulose in the digested wood [5]. 

The digestion of lignocellulose in lower termite guts has been the basic model for the industrial conversion of woody biomass via suitable microbes into bio-methane fuel and other co-products [3,4]. Based on the process suggested by the behavior of lower termites combined with our experience in the commercialization of dry, thermophilic anaerobic digestion over the past thirty-five years, we developed a process for the practical conversion of “wood wastes” into bio-methane fuel and other co-products [6,7,8]. The derived wood-to-methane industrial process consisting of five (3+2) steps or “W2M3+2” in its designation form follows the example of the termite natural process with certain important modifications and improvements to enhance the conversion efficiency and maintain process control. The first improvement in the conversion efficiency consists of the co-fermentation or co-digestion of wood with organic wastes with high nitrogen contents, namely, agricultural wastes in the form of pig manure or poultry litter. Pig manure and poultry litter contain roughly 10% nitrogen by weight in their dry states. This nitrogen in these manures is mostly found in the form of proteins, while about 20–30% has been mineralized in the form of ammonia. This results in a two-fold benefit: (a) it supplies an internal source of mostly organic nitrogen to the participating microbes vs. the external atmospheric supply of nitrogen in the termite system fixed by bacteria in the insect gut [2], and (b) it employs another organic waste product, i.e., animal manures, in a highly productive fashion. A second improvement consists of the thermophilic operation of the process vs. the mesophilic operation in termites to further increase the conversion rate. The initial “three” steps of the five-step industrial process are as follows: (a) maceration/size reduction in wood wastes into submillimeter-sized particles [9,10,11]; (b) homogenization and mostly aerobic upper thermophilic (up to 70 °C) hydrolysis of the macerated wood, with the manure relying primarily on the thermophilic fungi [10,11,12]; and (c) the fermentation of the hydrolyzed materials via bacteria and archaea under dry thermophilic (55 °C) conditions to yield biogas, namely, a mixture of about 60% methane and 40% carbon dioxide [7]. These “three” steps comprise the biological degradation/conversion of wood into a gaseous fuel. The last “two” steps, which do not occur in termites, are as follows: (d) membrane separation of the biogas into 99%+ pure bio-methane fuel [13] and (e) concurrent sequestration of the carbon dioxide [14] to generate additional/secondary bio-methane via its dry thermophilic anaerobic digestion with a requisite amount of hydrogen to be generated via wind or solar-powered water electrolysis [15,16,17].

A standardized, modular, industrial type plant has been developed, which processes 12,000 mt of wood wastes annually (air-dried at 80% TS) along with 24,000 mt of nitrogen-rich manures (diluted at 10% TS) to yield bio-methane (dry) per annum at a primary rate of 4.1 × 10^6^ Nm^3^ and at a secondary rate (from CO_2_ conversion with H_2_) of 3.1 × 10^6^ Nm^3^ [3,4]. No carbon dioxide is emitted, and either 2800 mt/yr of ammonium nitrate (50% TS) or 6200 mt/yr of ammonium sulfate (37% TS) is generated. Some 25 MW of wind power is required to supply energy, of which 95% is used for the production of hydrogen. Depending on the local and regional availability of the required wastes, a suitable number of such facilities could be built in a decentralized fashion.

## 3. The Thermophilic Fungi of Choice for Bio-Methane Production

As already discussed, wood size reduction is a key step in the decomposition of wood in the natural process and in the industrial process [3,4]. Wood particles of 0.2–2 mm in size are typically generated in the industrial process, whereby the cellulose crystallinity is disrupted. Moreover, the hugely increased surface area of wood immensely facilitates the exposure of cellulose and hemicellulose to the enzymes secreted by the thermophilic organisms that promote wood hydrolysis [9,10,11]. The hydrolysis process is driven primarily by thermophilic fungi [18,19,20], while thermophilic actinomycetes synergistically assist the fungi in the process [21,22]. In addition, about 20% to 30% of the nitrogen in the manures exists as ammonia, thereby enhancing the break-down of the wood particles by facilitating the solubilization of lignin in the “W2M3+2” industrial process [9,23,24].

**Figure 1:**
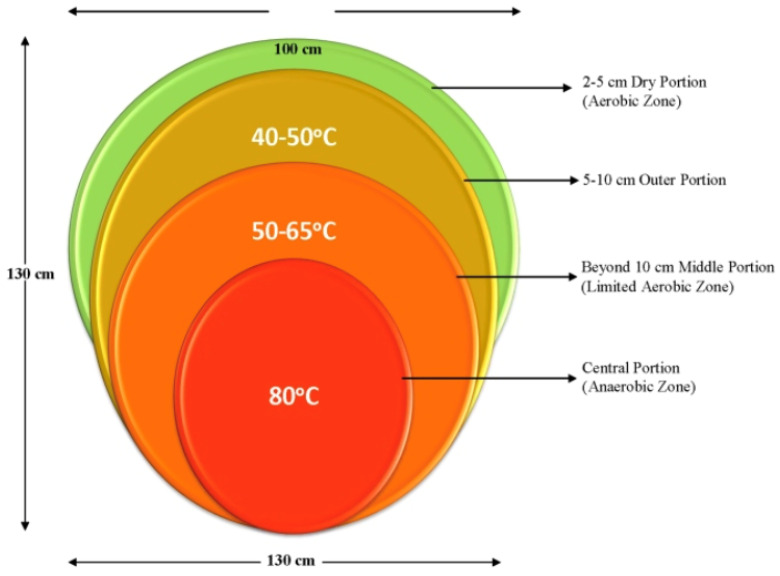
Cross-sectional temperature and oxygen/air distributions in a mushroom compost pile attained within 2 days of piling the wet materials [26], reproduced with permission from the Francis and Taylor Group).

Thermophilic fungi constitute the major component of the microflora that develop in heaped masses of plant material, piles of agricultural and forestry products, and other accumulations of organic matter, wherein a warm, humid, and mostly aerobic environment provides the basic conditions for their development [19,25]. The typical temperature and air distributions obtained in such an environment, reflected in a mushroom compost pile, is shown in Figure 1 [26]. The middle partially anerobic zone of the compost pile in Figure 1 would be similar in environmental conditions to the hydrolysis step of the “W2M3+2” industrial process.

Thermophilic fungi comprise only a very small number of species on the order of 30 to 40 out of about 50,000 recorded fungi species [23]. They have an optimum temperature for growth above 45 °C. Moreover, thermophilic fungi are the only known eucaryotic organisms to function in temperatures above 50 °C and perhaps as high as 70 °C [19,26]. Of course, the property of thermophily is much more widespread among other unicellular organisms such as blue-green algae (85 °C), actinomycetes (85 °C), and archaea (110 °C). Thermophily in fungi is obviously not as extreme as it is in eubacteria and archaea. We should also note that in general, the term of thermophily does not refer to a specific temperature that is applicable to all organisms, but rather to a temperature range that is applicable to a particular species, although it has a lower bound of 40 °C. In view of the intensive investigations that have been made of thermophilic algae, bacteria, and archaea since the 1950s, it is perhaps in the past twenty to thirty years that thermophilic fungi have begun to receive increased attention because of their potential applications [26]. The first thermophilic fungus species, *Mucor pusillus*, was described in 1886 [27]. W. Lindt discovered the first thermophilic fungus in 1886 growing on bread. H. Miehe and K Noack, two of the pioneers in the study of thermophilic fungi in the first two decades of the 20th century, were investigating the cause of temperature rise in hay. R. Emerson during the war years 1944-46, serving in the Guayule Rubber Extraction Research Unit of the U.S. Department of Agriculture in Salinas California, was engaged in a detailed examination of Guayule retting, i.e., a process in which the rubber producing shrub, *Parthenium argentatum*, was subjected to microbial action, including the thermophilic fungus *Humicola Insolens*, to raise the temperature of the shrub mass to temperatures up to 60 °C in order to yield better quality rubber [19]. In the first two decades of the 20th century, the number of studied thermophilic fungi species had risen to five, and toward the middle of the 1960s, it rose to thirteen species [19]. As it turns out, thermophilic fungi have been essentially observed since the discovery of their existence because of an interest in their activities regarding practical applications rather than because of trying to understand their taxonomy and morphology [26]. Thus, in the past three decades, researchers have isolated a dozen or so thermally stable enzymes out of about two dozen species of the known thermophilic fungi to utilize these enzymes in a variety of biotechnological applications [26]. The employment of thermophilic fungi in the hydrolysis of wood wastes to produce bio-methane is therefore no exception to the trend.

Because of the way thermophilic fungi have been discovered and studied for more than a century now, and particularly in the past half-century, there seems to be a confusion as to the true species of thermophilic fungi versus thermotolerant fungi [26]. Moreover, there exist variations among several species of thermophilic fungi with respect to their reproductive nature (sexual vs. asexual) that add further confusion to their classification and make the nomenclature even more cumbersome [26]. This also results in nomenclatural disagreements [25]. Nonetheless, we have been able to identify at least eleven thermophilic fungi species that can operate optimally at a temperature of 55 °C and possibly up to 70 °C. These fungi are found naturally in habitats where wood wastes, other cellulosic wastes, and manures exist [26,28]. These fungi then are of exceptional importance in the industrial wood hydrolysis and are the drivers of this process, and they are aided synergistically by thermophilic actinomycetes that are tolerant to even higher temperatures, as we will briefly discuss at the end of this section [29]. A summary of these eleven thermophilic fungi species, along with their optimal environmental temperatures, pH conditions, and respective natural habitats, is given in Table 1. It should be noted that three of the thermophilic fungi listed in Table 1, namely, *Chaetomium thermophile* var. *coprophile*, *Sporotrichum thermophile*, and *Thermoascus aurantiacus*, have been reported over half a century ago to be two to three times as active in hydrolyzing cellulose as one of the most vigorously cellulolytic mesophile fungus, *Trichoderma viride*, yet it is only now that these fungi, along with other thermophilic fungi, are employed in an industrial setting to convert wood wastes into bio-methane via the “W2M3+2” process [30,31].

In the wood hydrolysis step of the industrial operation, “W2M3+2”, several of these species of thermophilic fungi are believed to operate synergistically along with thermophilic actinomycetes to break down cellulose and hemicellulose into simple sugars, depending on the specific compositions of the processed wastes. For example, *Thermomyces lanuginosus*, also named or known in earlier studies as *Humicola lanuginosus*, which, by itself, cannot metabolize cellulose, has shown profuse growth in mixed cultures with the cellulolytic fungus, *Chaetomium thermophile* [20,32]. Another vigorous cellulose-degrading fungus is *Thermoascus aurantiacus*. In fact, all eight thermophilic fungi species included in Table 1 that grow on wood chips (*Chaetomium thermophile* var. *coprophile*, *Chaetomium thermophile* var. *dissitum*, *Humicola grisea* var. *thermoidea*, *Humicola insolens*, *Scytalidium thermophilum*, *Talarmyces emersonnii*, *Thermoascus aurantiacus*, and *Thermomyces lanuginosus*) are in one way or another the prime fungal organisms that can degrade wood. The simple sugars generated in the hydrolysis step are then converted in the co-fermentation (second) step of the industrial process via thermophilic acetogenic bacteria into acetate, which, in turn, is converted to bio-methane and carbon dioxide by thermophilic methanogenic archaea. We may also note that thermophilic fungi are not known to have any special nutritional requirements as they can grow on simple media containing carbon, nitrogen, and mineral (K, Mg, Fe, Zn, and Cu) salts [26]. They are also mostly autotrophic. The ideal carbon (C) to nitrogen (N) growth ratio is twenty to one [26]. This is also the ideal C to N ratio for the fermentation step to optimize methane production, and it has been the basis of the selection of the respective amounts of wood wastes and manures in the “W2M3+2” industrial process, as indicated in the previous section [7,8,22]. It has been suggested that thermophilic fungi prefer non-ammonium-based nitrogen sources. Indeed, the supply of nitrogen in the nitrogen-rich manures is in its preponderance (70% to 80%) in a protein form, with the remainder being mineralized inorganic nitrogen in the form of ammonia. Another crucial, but usually overlooked, growth parameter of thermophilic fungi is the ratio of carbon (C) to phosphorous (P) [26]. The desired value of this ratio should be in the range of 120:1 to 240:1 to ensure optimal growth and is readily met in the industrial process as the nitrogen-rich manures are also phosphate-rich [7,8,22].

An important environmental factor of growth for thermophilic fungi is the pH factor, as suggested in Table 1. The pH of the environment exerts a profound influence on the transport of nutrients, solubility of nutrients, enzymatic reactions, and availability of specific metallic ions that may form soluble or insoluble complexes at a particular pH value. For example, metals like Mg, Fe, Ca, and Zn are available to a fungus at low pH values and become insoluble at higher pH values. Obviously, the pH preferences of individual fungi vary. Most thermophilic fungi tend to grow in the acidic range and/or a somewhat alkaline one, but they are also tolerant to a broad range of pH values from 4 to 8 [26]. These pH ranges are also consistent with the pH occurring in the hydrolysis step in the industrial process. As is to be expected, the pH in the hydrolysis step in the industrial process is highly dynamic in value and distribution. It varies across the reactor vessel from acidic to slightly alkaline and is affected by the biodegradation of the carbon and nitrogen sources. Optimal values of the pH are in the range of 5.5 to 8.9 for the fungal activities and from 6.0 to 7.5 for the bacterial activities [22,33].

The effect of oxygen on the growth of thermophilic fungi is obviously another critical environmental parameter, perhaps next to temperature in importance. The fermentation step of the industrial process is strictly anaerobic. However, in state-of-the-art dry digestion, and in anaerobic digestion systems such as ours, metered amounts of air are introduced into the digester such that the sulfides are oxidized to sulfates that remain in the liquid phase, thereby ensuring that the generated biogas is nearly free of hydrogen sulfide. The wood hydrolysis step where the thermophilic fungi constitute the primary microbial conversion agent varies from being aerobic to partially anaerobic across the reactor vessel. The consensus appears to be that fungi in general and thermophilic fungi more specifically are aerobic [26,28,33]. However, there is sufficient evidence to suggest that thermophilic fungi do not stop their growth in reduced oxygen environments, and in fact, they can survive well in anoxic conditions. For example, the pioneering investigation of the fungus *Thermoascus aurantiacus* indicated that its respiratory quotient (CO_2_/O_2_) remained practically the same, even at very low concentrations of oxygen, and that the fungus could withstand anaerobic environments continuously for 8 days without the loss of viability [34]. A study of the fungus *Humicola insolens* reported better growth under anaerobic or microaerobic conditions rather than aerobic ones [35]. Another study observed that the perfect (ascocarpic) condition of the fungus *Penicilllium duponti* vs. the fungus *Talaromyces dupontii* was initiated only under anaerobic conditions [19,28,36]. Studies on the requirements of oxygen in thermophilic fungi are limited, but it is believed that most of them require at least 0.2% oxygen for trace growth and 0.7–1.05% oxygen for moderate growth [26]. Obviously, the amount of oxygen in the hydrolysis step of the industrial process, “W2M3+2”, is a controlled parameter as it is in the fermentation step, albeit for entirely different reasons [3,4].

As it has been mentioned already in several instances, the wood hydrolysis step of the industrial process, while dominated by thermophilic fungi, is also aided by thermophilic actinomycetes. Actinomycetes are bacteria that grow in the form of mycelia, like that of fungi, with the difference being that bacterial mycelia have a typical dimeter of 1 mm vs. fungal mycelia, which have a typical diameter of 5 mm [37]. The name Actinomycetes is derived from the Greek words “aktin” for ray and “mykes” for fungus because of the morphological shape of these bacteria and their resemblance to fungi. It is also interesting to note that while the first antibiotic to ever be isolated was “Penicillin” from the mesophilic fungus *Penicillium notatum* in 1928 but did not go into production until 1942, two other antibiotics were isolated and crystalized from mesophilic actinomycetes: “Actinomycin” from *Streptomyces antibioticus* and “Streptomycin” from *Streptomyces griseus* in 1940 and 1942, respectively. Many more antibiotics have been derived from actinomycetes since then. Thermophilic actinomycetes consist of species in several genera such as *Thermoactinomycetes*, *Thermomonospora*, *Microbispora*, *Sacharopolyspora*, and *Strepotmycetes* [37,38]. For example, the thermophilic actinomycetes species, *Strepotsporangium*, is one of the many cellulose-degrading streptomyces [37,38]. Thermophilic actinomycetes are aerobic, Gram-positive, and have optimal growth temperatures of up to 75 °C, although they can survive at much higher temperatures [36]. While the aid of the thermophilic actinomycetes to thermophilic fungi in degrading cellulose has been known for more than eighty years, other thermophilic bacterial genera, i.e., non-actinomycetes, aiding in the process, directly or indirectly, have been identified in recent decades [21,39,40]. Thus, the thermophilic bacterium species, *Thermus thermophilus*, in the aerobic, Gram-negative genus, *Thermus*, grows in different piles of cellulosic and nitrogen-rich wastes (garden and kitchen wastes, sewage sludge) with optimal growth between 65 °C and 75 °C [37,39]. Moreover, a variety of bacteria that are homologous to the thermophilic bacteria species *Bacillus schlegelii* and *Bacillus stearothermophilus* in the Gram-negative genus *Bacillus* have been isolated in hot composts [37,40]. These thermophilic bacteria have an optimum growth at 70 °C to 75 °C, but under microaerophilic conditions of 5 kPa oxygen [40]. Undoubtedly, many more microbial species that have not yet been isolated and studied would exist in the wood hydrolysis step, because they can grow in that environment and consequently support the process in a symbiotic manner. Recent reviews on the biodegradation of wood, albeit in different environments than that of the “W2M3+2” process, confirm the extensive symbiosis of fungi with bacteria [41,42]. Forest ecosystems are estimated to store 861 Pg (Peta-grams) of carbon, of which about 73 Pg represent deadwood. Globally, wood decomposition into carbon dioxide is variously estimated to range from 2.1 to 11 Pg of carbon per year. The return of carbon dioxide into the atmosphere via wood decomposition is thus similar in magnitude to that from the current global fossil fuel combustion amounting to 9.5 Pg of carbon annually. (Note—1 Pg is equal to 1 billion metric tons.) It is interesting to note that the conversion of wood wastes globally per the “W2M3+2” process would reduce the emissions of CO_2_ globally by an estimated amount of 2.6 billion metric tons of carbon per year just from not allowing the normal deadwood decomposition to occur and by thinning forests to reduce fires [4]. The estimated potential of waste wood to be collected annually to be processed into bio-methane is about 5.3 billion metric tons, of which about 2.6 billion tons is carbon, given that 48–50% of dry wood biomass is carbon by weight. The avoided carbon equivalent of annual greenhouse gas emissions from the utilization of pig manure and poultry litter to co-digest with the available waste wood is estimated to be 0.7 billion metric tons and 0.6 billion metric tons, respectively [43]. Thus, the application of the “W2M3+2” process technology would globally eliminate, at a minimum, some 3.9 billion metric tons of carbon annually. This is a quite significant amount, representing about 41% of the current fossil fuel carbon emissions of 9.5 billon metric tons per year [39,40]. It may take a period of about 20 to 30 years for the annual collection of most naturally occurring deadwood along with forest thinning to be established globally to sequester emissions of carbon dioxide and produce renewable natural gas. During the same time, the use of fossil fuels for power generation could be supplanted by the increased penetration of renewable wind and solar electricity, thereby curtailing the overall greenhouse gas emissions from fossil fuels. The global implementation of the “W2M3+2” process has the potential to supply over one-fourth of the current world energy use and 105% of the current world consumption of natural gas [4]. Assuming, for example, that this occurs by 2050, and at the same time, some 50% of electricity production globally is based on wind and solar power, then the fossil fuel carbon footprint would be diminished to at least one-half and likely to one-third its present value, given that global power generation relies heavily on coal combustion and that “W2M3+2” bio-methane production can essentially replace most if not all of fossil and natural gas use. Thus, the global implementation of the “W2M3+2” process could, by 2050, totally offset the emissions from fossil fuels at that time and ultimately lead to a net carbon dioxide removal from the atmosphere.

Based on our knowledge of the anaerobic co-digestion of organic wastes as well as our current understanding of the wood to bio-methane conversion in lower termites, it is to be expected that the industrial wood hydrolysis step in the “W2M3+2” process relies on a multitude of thermophilic fungi that operate synergistically with certain bacteria and perhaps even other microorganisms to be identified over time.

## 4. Enhancing Thermophilic Fungi for Increased Bio-Methane Production

The ability to use complex carbon sources and thrive at high temperatures is the most important characteristic of thermophilic fungi. Consequently, thermophilic fungi are uniquely able to bio-degrade wood wastes, specifically cellulose and hemicellulose, into simple sugars that subsequently can be fermented into bio-methane. However, these thermophilic fungi must find themselves in the proper environmental conditions to function optimally. And that is exactly what the design of the “W2M3+2” industrial process strives to attain [3,4]. Moreover, the “W2M3+2” industrial process relies on intact fungi for its successful operation, contrary to unsuccessful efforts in the past two decades to just grow suitable fungal enzymes for a variety of applications [26].

A naturally occurring question is whether the wood hydrolysis ability of the thermophilic fungi of choice, as shown in Table 1, can be enhanced and further improved. The obvious characteristic to be potentially improved is the optimal operational temperature to reach the 70 °C upper boundary of thermophily. A secondary improvement could be their ability to grow under partially anaerobic conditions.

A cursory examination of Figure 1 clearly demonstrates that thermophilic fungi are thermotolerant up to at least 80 °C and can survive under anaerobic conditions. The optimal temperature data shown in Table 1 reflect the lower temperature range of thermostable cell-free enzymes produced by the respective fungi [26,28]. On the other hand, the optimal operational temperature of these fungi themselves is uncertain, but it is presumed to be lower by as many as 10 °C, on average, compared to the respective values shown in Table 1 [26]. As already indicated, it is desired that the hydrolysis step of the industrial “W2M3+2” process occurs in an operational temperature, which serves as a process parameter, of at least 55 °C and ideally closer to 70 °C to maximize the biodegradation of the wood and all cellulosic components in the feedstock material. In addition, maintaining the high biodegradation rate in a reduced oxygen environment is a second desired characteristic, albeit of secondary importance, because a certain degree of aeration always exists in the hydrolysis step of the “W2M3+2” industrial process.

One potential mechanism to increase the upper operational temperature limit of a thermophilic fungus and perhaps even maintain its growth in a reduced oxygen environment could be the presence of another organism inside the fungus. In other words, the microbiome of the fungus will be modified by another smaller organism that lives in a symbiotic relationship in the host fungus. Given the limited knowledge at the present time regarding the interaction of fungi with other microorganisms such as bacteria and viruses, we use the term, symbiosis, to generally describe all situations whereby a particular microorganism either works with a fungus synergistically (exo-symbiosis) or else has become a part of the fungal biome (endosymbiosis). This organism could be a bacterium or a virus. It is gradually becoming established that symbiosis with microbes represents an integral part of the survival and well-being of complex organisms such as humans [44]. But bacteria have viruses within them (nano-biome), and it is envisioned that even viruses can contain smaller viruses within them (pico-biome) [45]. Symbiosis is a ubiquitous feature of life, and it no longer makes sense to study individual organisms; rather, the relationships between living organisms should be studied. There are several examples of the effects of microbes that live within fungi. Fungal viruses or mycoviruses can modulate plant–fungal symbiosis. The best-known example is the hypo virus that attenuates the virulence of the chestnut blight fungus, *Cryphonectria parasitica* [46]. At the other end, a plant–fungal symbiosis between a tropical panic grass from geothermal soils, *Dichanthelium lanuginosum*, and the fungus *Curvularia protuberata* allows for both organisms to grow at high temperatures in thermal springs [47]. However, it turns out that the fungus *Curvularia protuberata* is also infected with a virus in a three-way symbiosis to confer its thermal tolerance not only to itself but also to the plant [48]. When grown without the virus, neither the plant nor the fungus can survive high temperatures. But the microbiome of the fungus populated with the virus allows the fungus and the plant, into whose leaves the fungus lives, to survive temperatures up to 65 °C. In another example of how the fates of fungi are intertwined with other microbes, the rice blast fungus *Rhizopus microsporus* associates itself with a bacterium that produces the key toxins causing the disease but also allows it to produce spores, i.e., reproduce asexually [49].

It is evident, then, that the microbiome of a thermophilic fungus populated with suitable bacteria and/or viruses could modify its behavior such that it can grow in temperatures near or at the upper thermophilic range of 70 °C. In fact, no known studies exist where the microbiome of thermophilic fungi has ever been examined to determine whether bacteria or viruses are present and can account for the observed range of temperature growth in any fungal species. Moreover, the same or different bacteria and/or viruses in the fungal microbiome could affect the sexual (w/o spores) or asexual (w/spores) types of reproduction of a thermophilic fungus in which the sexual stage has not been discovered so far or is observed infrequently such that it can grow in varying oxygen environments [28,36]. An interesting observation is related to the occurrence of thermophilic fungi in the aquatic sediments of lakes and rivers, where low temperatures (6–7 °C) and very low oxygen levels (average 10 ppm, <1 ppm in at a depth of 31 m near the bottom of lakes) exist [26,50]. No explanation of that observation has been given, although a microbiome enhancement of the fungi by certain bacteria and/or viruses could be a plausible explanation that requires verification. A final observation, which may prove to be relevant in the future to increase the efficiency of the controlled conversion of wood wastes to bio-methane using the “W2M3+2” industrial process, has to do with the discovery of fungal DNA in the sapwood of healthy trees [51]. This suggests that deadwood from these trees would be susceptible to a faster natural biodegradation to carbon dioxide and perhaps also a more efficient controlled biodegradation to bio-methane in the presence of suitable fungal species.

Obviously, significant research efforts must be carried out to elucidate and implement the appropriate microbiome enhancements of thermophilic fungi. Most likely, these microbiome-enhanced thermophilic fungi already exist in natural environments and are waiting to be identified as such by researchers. We may mention that it is also well understood now by the scientific community that only a small fraction of about 1% of microbes, including fungi, have been cultured in the laboratory, thereby necessitating the development of alternative faster and less expensive methods of assessing microbial diversity in different physiological niches [26].

## 5. Discussion and Conclusions

An industrial two-step process, comprising hydrolysis and fermentation, and designated as “W2M3+2”, has been developed to mimic the natural conversion of wood into methane by the lower termites, albeit in a thermophilic temperature regime. The hydrolysis step of this process relies on thermophilic fungi aided by thermophilic actinomycetes. Of the known 30 to 40 thermophilic fungi species, some eleven species have been identified as likely candidates to promote the hydrolysis of wood under controlled conditions. The operating temperatures of the process are in the 55 °C to 70 °C range, with the upper value of the range being desirable for the hydrolysis step, while the lower value of the range at 55 °C is optimal for the fermentation step. Most of the known thermophilic fungi that are relevant for the industrial process have an optimal growth temperature above 55 °C but below 70 °C. It is therefore suggested, based on experience with other microorganisms, that future research should address the incorporation of suitable bacteria and/or viruses into the microbiome of selected thermophilic fungi to bring the optimal operating temperature of these fungi closer to 70 °C. In addition, the elucidation of the conditions under which certain thermophilic fungi can operate in partially anaerobic environments should be better understood. These investigations on thermophilic fungi would lead to long-term further improvements in the conversion efficiency of the already developed “W2M3+2” industrial process. The utilization of thermophilic fungi in a synergistic operation with other thermophilic organisms comprising bacteria and archaea in an industrial setting undoubtedly leads to the conversion of wood wastes and other cellulosic wastes, along with nitrogen-rich organic wastes, into bio-methane fuel and results in greenhouse gas emission offsets comparable to those currently produced from the utilization of fossil fuels. These greenhouse gas emission offsets could be further improved by the successful realization of the proposed research efforts outlined in this report.

## Figures and Tables

**Table 1:** Thermophilic fungi of relevance in the controlled hydrolysis of wood wastes mixed with nitrogen-rich organic wastes in the temperature range of 55 °C to 70 °C prior to the co-fermentation of these wastes into bio-methane.

No.	Fungus	Optimal	Optimal	Natural Habitat
		pH	Temp. °C	
1.	*Chaetomium thermophile* var. *coprophile*	6.0	60	Wood chips
2.	*Chaetomium thermophile* var. *dissitum*	5.0	55	Wood chips
3.	*Humicola grisea* var. *thermoidea*	4.0–4.5	60	Wood chips
4.	*Humicola insolens*	5.6	50	Wood chips
5.	*Myceliophthora thermophiia*	6.6	60–70	Soil
6.	*Scytalidium thermophilum*	5.5	65–70	Poultry litter, wood chips, soil
7.	*Sporotrichum thermophile*	5.4	65	Wood chips
8.	*Talarmyces emersonnii*	4.0–4.5	70	Compost, soil, wood chips
9.	*Talaromyces dupontii*	4.5	70	Manures, hay, soil, ag residues
10.	*Thermoascus aurantiacus*	4.5–5.5	60–65	Hay, sawdust, wood chips
11.	*Thermomyces lanuginosus*	4.4–6.6	70	Ag residues, pig manure, soil
